# Supplementary material for: Inhibitory Effect of Isoliquiritigenin in Niemann-Pick C1-Like 1-Mediated Cholesterol Uptake
Source: Molecules. 2022 Nov 3;27(21):7494. doi: 10.3390/molecules27217494 (PMC9654431; doi:10.3390/molecules27217494)
Supplement: Supplementary file 1 [file molecules-27-07494-s001.zip › molecules-1992530-supplementary.pdf]

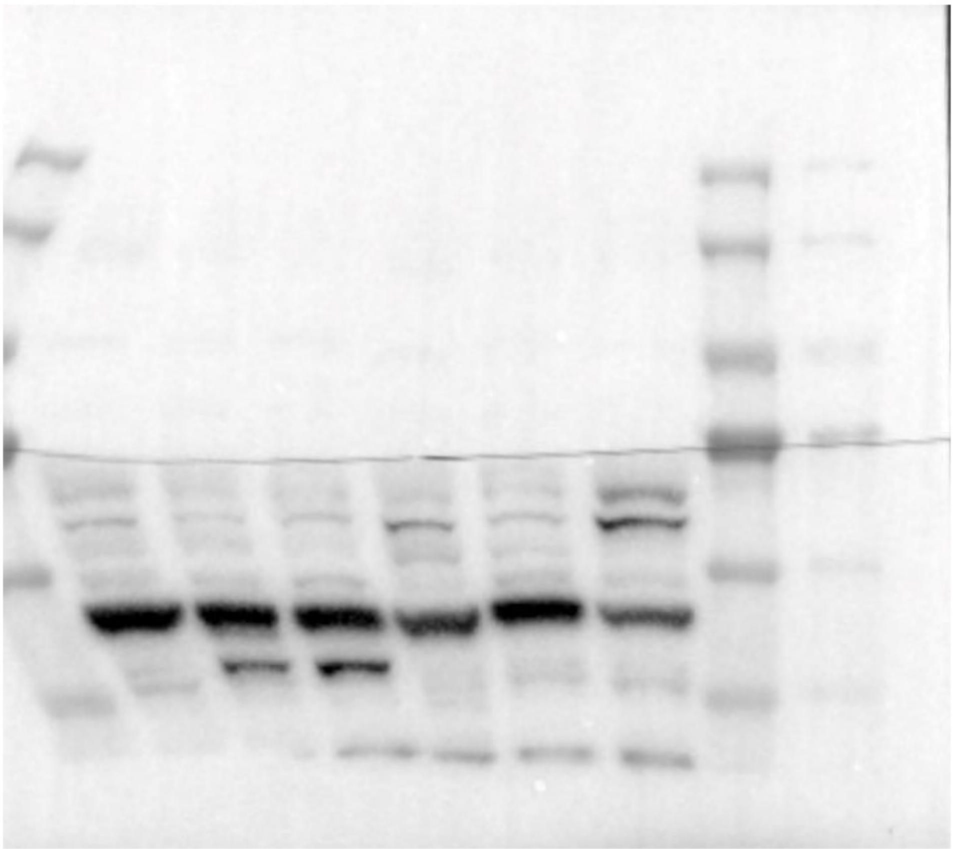

Figure S1. The full PVDF membrane diagram of Figure 3.

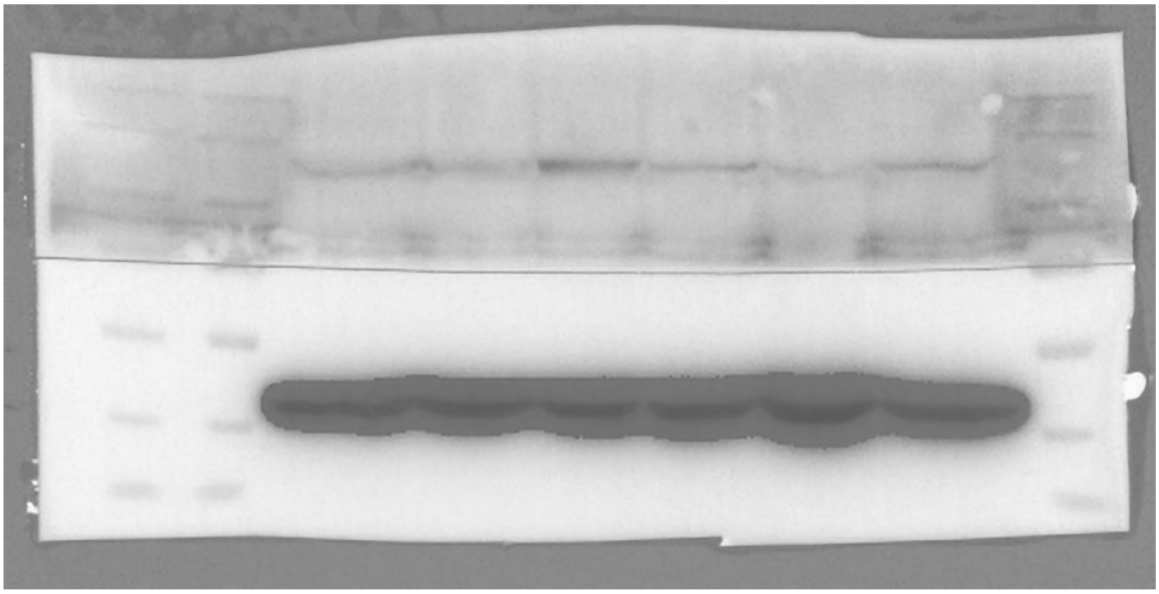

Figure S2. The full PVDF membrane diagram of Figure 8.
